# Wild whale faecal samples as a proxy of anthropogenic impact

**DOI:** 10.1038/s41598-021-84966-4

**Published:** 2021-03-12

**Authors:** Marianna Marangi, Sabina Airoldi, Luciano Beneduce, Claudio Zaccone

**Affiliations:** 1 Department of the Sciences of Agriculture, Food, Natural Resources and Engineering (DAFNE), University of Foggia, Via Napoli 25, 70122 Foggia, Italy; 2 Tethys Research Institute, Viale G.B. Gadio 2, 20121 Milan, Italy; 3 Department of Biotechnology, University of Verona, Strada Le Grazie 15, 37134 Verona, Italy

**Keywords:** Ecology, Microbiology, Chemistry

## Abstract

The occurrence of protozoan parasite, bacterial communities, organic pollutants and heavy metals was investigated in free-ranging species of fin (*Balaenoptera physalus,* n. 2) and sperm (*Physeter macrocephalus,* n. 2) whales from the Pelagos Sanctuary, Corsican-Ligurian Provencal Basin (Northern-Western Mediterranean Sea). Out of four faecal samples investigated, two from fin whales and one from sperm whale were found positive to *Blastocystis* sp. A higher number of sequences related to Synergistetes and Spirochaetae were found in sperm whales if compared with fin whales. Moreover, As, Co and Hg were found exclusively in sperm whale faecal samples, while Pb was found only in fin whale faecal samples. The concentration of both PAH and PCB was always below the limit of detection. This is the first report in which the presence of these opportunistic pathogens, bacteria and chemical pollutants have been investigated in faecal samples of free-ranging whale species and the first record of *Blastocystis* in fin and sperm whales. Thus, this study may provide baseline data on new anthropozoonotic parasite, bacterial records and heavy metals in free-ranging fin and sperm whales, probably as a result of an increasing anthropogenic activity. This survey calls for more integrated research to perform regular monitoring programs supported by national and/or international authorities responsible for preservation of these still vulnerable and threatened whale species in the Mediterranean Sea.

## Introduction

**Figure 1:**
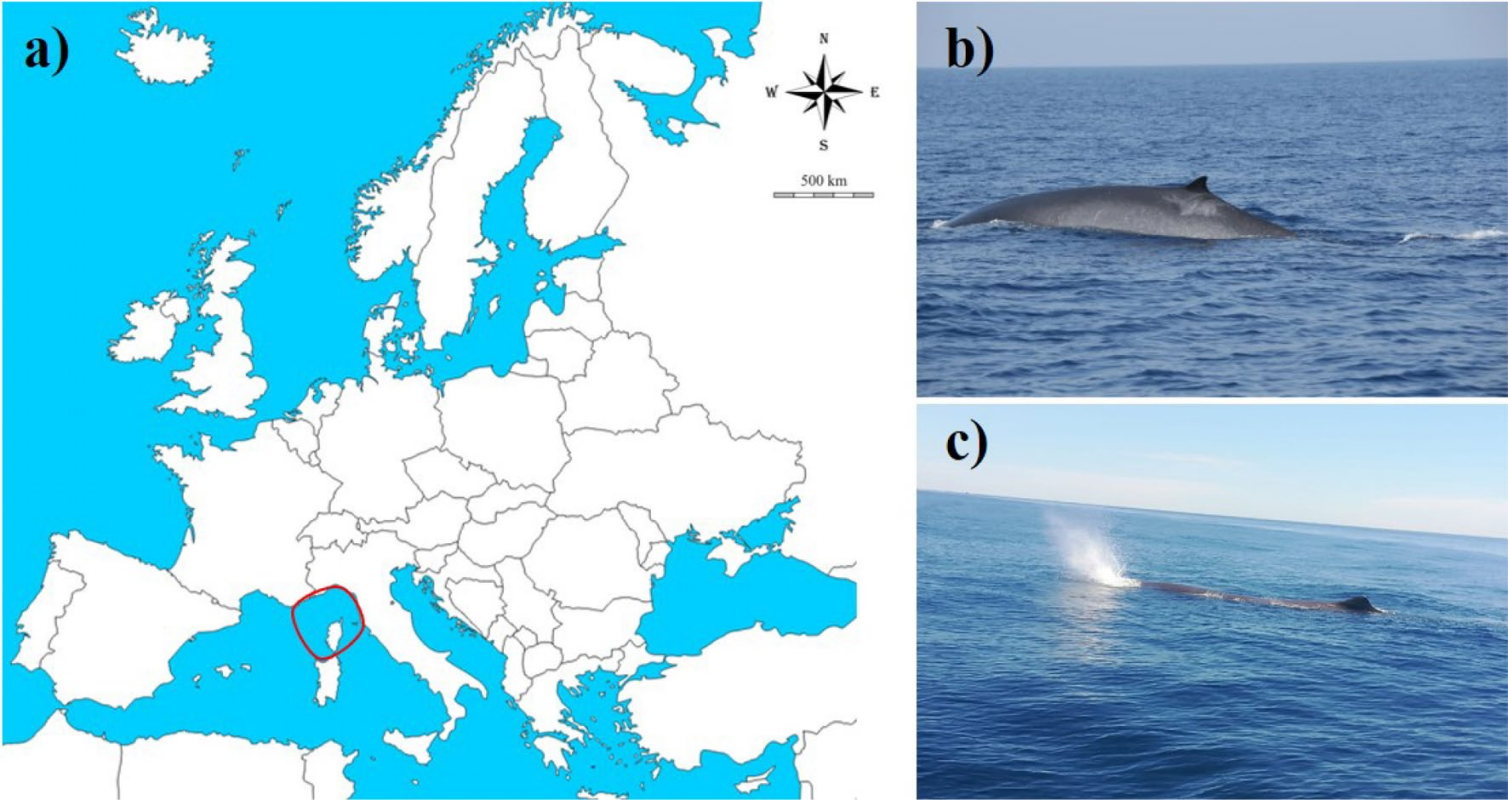
Fin and sperm whale faeces sampling. (**a**) Sampling area. The red circle indicates the Pelagos Sanctuary, Corsican-Ligurian Provencal Basin. The map was created using QGIS v. 2.14 software (http://www.qgis.org/it/site/). (**b,c**) Details about the fin whale and the sperm whale in the wild, respectively (photos by M.M. and Tethys Research Institute).

The Mediterranean Sea, the largest and deepest enclosed sea on Earth, represents an ideal habitat for different species of marine animals, contributing to a great biodiversity at global level as well^1^. The Northern-Western Mediterranean Sea, in which an International Marine Protected Area (Pelagos Sanctuary, Corsican-Ligurian Provencal Basin) is included (Fig. 1), is known to be regularly inhabited by eight species of cetaceans^2,3^. Among them, the Mediterranean subpopulations of fin (*Balaenoptera physalus*) and sperm (*Physeter macrocephalus*) whales are ranked as vulnerable and endangered species, respectively, by the International Union for Conservation of Nature Red List^4^. In the Pelagos Sanctuary area, these species are particularly exposed to both infectious diseases and anthropogenic activities that represent a potential threat to their long-term survival^5^. Parasites, bacteria, as well organic and inorganic pollutants, are considered among the main causes of whale death^6,7^ or factors predisposing them to other pathologies^8,9^.

In detail, whales can be affected by a wide range of naturally occurring endo- and ectoparasites, most of which are highly pathogenic^10^. Furthermore, several parasites species have gained importance as opportunistic pathogens in the marine environment. The introduction of these new emerging and neglected parasites (e.g., protozoan parasites), most of which transmitted by ingestion of contaminated food and water, probably occurs through terrestrial contamination and is generally due to intense human activities^6,11,12^. Whales, like other mammals, host diverse bacterial and archaeal symbiont communities, that play important roles in digestive and immune system functioning. Erwin et al.^13^, investigating pigmy and dwarf sperm whales (*Kogia sima*) gut microbiomes, showed that host identity plays an important role in structuring cetacean microbiomes, even at fine-scale taxonomic levels. Therefore, understanding whether the gut microbiota could be also affected by diet, environmental pollution, presence of gut pathogens and, in turn, influence the health status of cetaceans^14^, is of paramount importance. Finally, over the last decades, there have been numerous studies demonstrating elevated exposure of marine mammals to both persistent organic pollutants (including polychlorinated biphenyls (PCBs) and polycyclic aromatic hydrocarbons (PAHs)), and heavy metals (e.g., Hg, Pb, As), which are generally associated to increased mortality, incidence of diseases and/or impaired reproduction^15–18^.

Fin and sperm whale populations strongly need a variety of conservation and monitoring measures, which would benefit of physiological and pathophysiological information, such as pathogen infections or chemical pollutants, gathered from free-ranging instead of stranded or caught animals. Moreover, due to sample collection difficulties, some aspects, including the gut microbiomes of apparently healthy, wild individuals, remain largely unexplored.

To our knowledge, no studies that includes analyses on parasites, bacterial infection and chemical pollutants in faecal samples have been carried out on whales in the wild so far. One of the reasons is that whales spend most of their time underwater and remain on the surface only for a very short time; moreover, collecting samples from free-ranging whales is cost effective and requires trained personnel and many days in the open sea just to recover few samples. Finally, biopsy sampling method, considered since the 1990s as one of the most common and less invasive tissue sampling technique^19^, remains a practice not easy to manage and only useful to get a selective type of information^10^. For all these reasons, data on parasites and bacteria fauna, as well as on organic and inorganic pollutants, are extremely rare for free-ranging whales.

The aim of this work was to establish a background concentration for parasites, bacteria, organic pollutants and heavy metals in free-ranging fin and sperm whales from the Pelagos Sanctuary area (Fig. 1 and Table 1) by using a multidisciplinary and non-invasive approach, in order to provide insights on the health status of these vulnerable and endangered species within their natural habitat.

**Table 1:** Sample ID, sampling date, GPS coordinates and size of the investigated fin and sperm whales.

Sample ID	Species	Sampling date	Latitude	Longitude	Size (m)
#1	Fin whale	04 September 2018	43.5773	7.8055	11–13
#2	Fin whale	21 September 2018	43.6000	7.8988	11–13
#3	Sperm whale	16 June 2019	43.5509	7.7712	13–15
#4	Sperm whale	25 July 2019	43.7300	7.9335	11–13

## Results

### Coprological and molecular analyses

**Figure 2:**
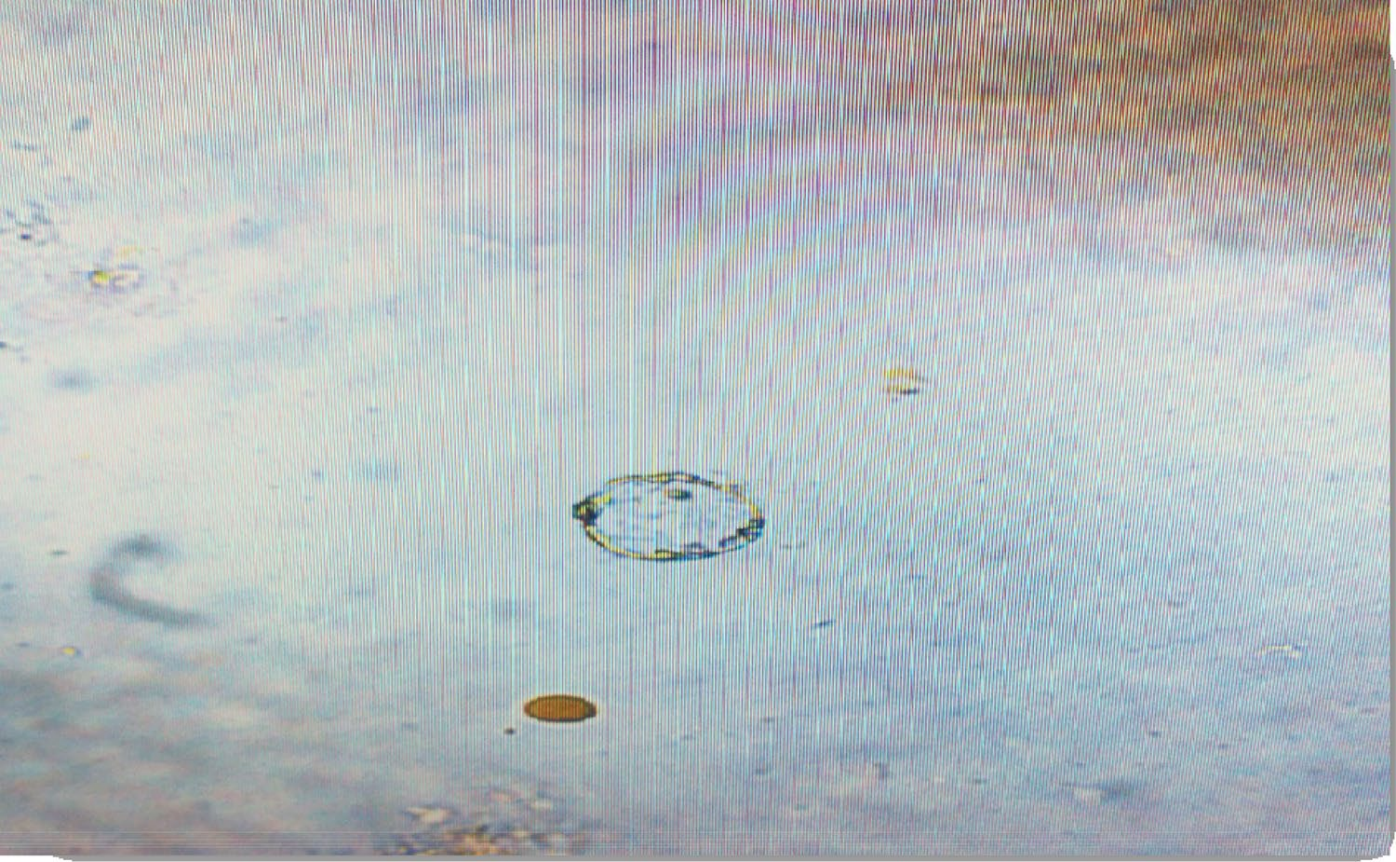
Cyst of *Blastocystis* in the faecal sample of fin and sperm whales (× 40 magnification) (photo by M.M.).

Cysts of *Blastocystis* (Fig. 2) were identified in the faecal samples of fin and sperm whales by coprological examination. No other cysts/oocysts/eggs referred to other parasites were found. The DNA samples subjected to *SSU-rDNA* PCRs were successfully amplified and, after sequencing, good quality sequences of about 600 bp were obtained. Aligned sequences revealed the absence of any stop codons with 100% identity each other. The alignment with the homologous sequences of *Blastocystis* sp. available in GenBank showed a mean percentage identity of 99% with *B. hominis*. The phylogenetic analysis using *SSU-rDNA* data sets were concordant in confirming the identity of the specimens examined here as *Blastocystis* and the sequences cluster with the *Blastocystis* ST3 in a monophyletic group distant from the other *Blastocystis* subtypes.

### Bacterial pathogens

Among pathogens of human and zoonotic origin, *Salmonella* spp. and enterohaemorrhagic *E. coli* were checked in all samples by PCR. They were found to be negative in all cases. The absence of these pathogens has been confirmed by high throughput sequencing analyses, since no homologous sequence at species nor genus level was found. About fish pathogens, that commonly may cause pathologies to marine mammals^20^, *Brucella* spp., *Staphylococcus* spp., *Leptospira* spp. *Nocardia* spp. and *Actynomices* spp. were not found in the specimens surveyed. A relative low number of sequences related to marine mammal opportunistic pathogens were found and, in all cases, they were exclusive of one species. In detail, *Mycobacterium* and *Fusobacterium* spp. were found only in sperm whale, whereas *Erysipelothrix* spp. and *Helicobacter* spp. only in fin whale.

### Microbial community composition

**Figure 3:**
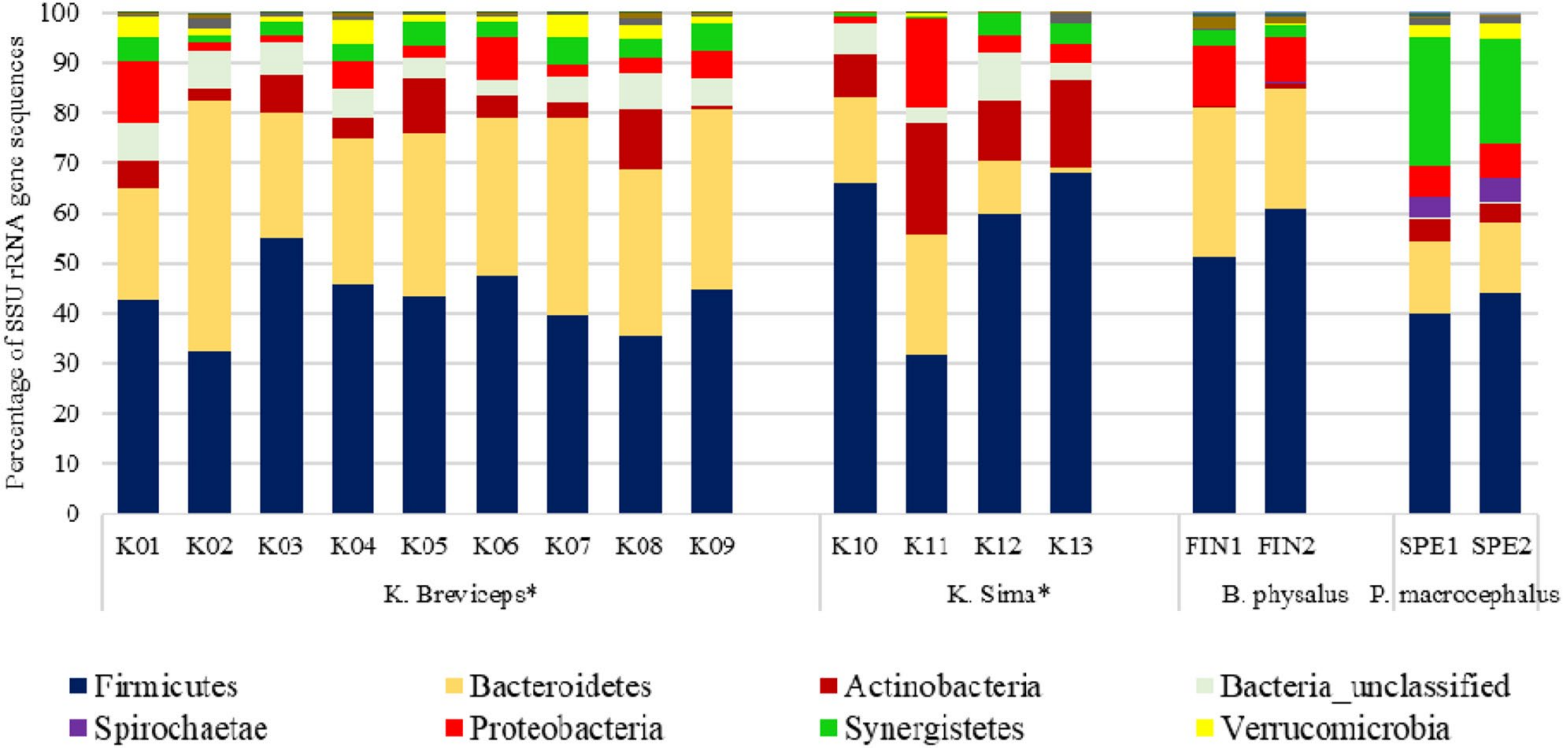
Phylum-level composition of gut microbiomes of whales analysed in this study, compared with previous data from other toothed whales (* data from Erwin et al.^13^).

**Figure 4:**
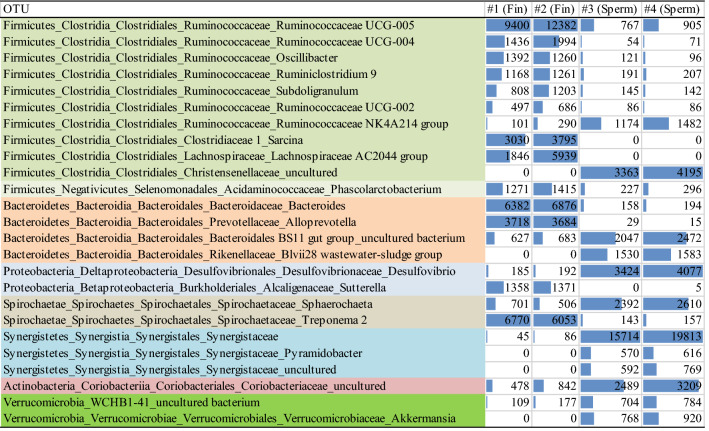
Genus level classification of the most representative (> 0.1% of total reads) and significantly different (Kruskal–Wallis, *p* < 0.05) OTUs found among the two whale species sampled. Different colours represent the different phyla; Histogram bars represent the abundance level of each OTU (quartile scale).

Figure 3 reports the phylum level distribution of bacterial taxa in the sampled whale faeces. A clear host specific pattern is visible between the two different species. In both cases Firmicutes and Bacteroidetes were the dominant phyla, and this finding agrees with previous studies^13,21,22^. Sperm whales were characterized by a higher number of sequences related to Synergistetes and Spirochaetae, as well as Verrucomicrobia and Actinobacteria, if compared with fin whales. Figure 4 shows in detail the most relevant differences among the gut microbiome of the sampled whales. The OTUs with an average number of reads > 0.1% threshold and that were significantly different (*p* < 0.05) were included in this comparison.

In the Firmicutes phylum, fin whales had higher number of *Ruminococcaceae*, *Lachnospiraceae*, *Oscilligranullum*, *Ruminiclostridium*, *Subdoligranulum*, while the most representative Firmicutes in sperm whales belonged to *Christensellaeae* and a different clade of *Ruminococcaceae* (NK4A214 group).

Among Bacteroidetes, *Bacteroides* spp. and *Alleloprevotella* spp. were typical of fin whale microbiomes, while sperm whales were dominated by *Rickenellaceae* and a different clade of Bacteroidales belonging OTU. Also, the most relevant *Proteobacteria* were different between the two species, being *Sutterella* spp. representative of fin whales and *Desulfovibrio* spp. of sperm whales.

About the Spirochaetae phylum, in sperm whales most sequences belonged to *Sphaerocheta* spp., while in fin whales *Spirochaeta* spp. and *Treponema* spp. were the most representative genus.

Among sequences belonging to Synergistetes, that were almost exclusively found in sperm whales, only one OTU was identified to the family level as *Synergistaceae* and one OTU at genus level as *Pyramidobacter* spp. Verrucomicrobia were highly prevalent in sperm whales compared to fin whales. Among the retrieved OTUs in sperm whales, one showed high similarity with uncultured not taxonomically defined bacterium and a second one was identified at genus level as *Akkermansia* spp. Finally, sperm whales also showed higher number of actinobacteria. More specifically the OTUs found belonged to the *Coriobacteriaceae* family.

### Elemental composition, and occurrence of organic and inorganic pollutants

The elemental composition of faecal samples strongly differed in the two whale species. In particular, faecal samples from sperm whales were characterized by a significantly higher average concentration of carbon (49.8 vs. 21.5%), nitrogen (13.6 vs. 3.4%) and sulphur (2.5 vs. 0.8%).

The concentration of 16 United States Environmental Protection Agency (US EPA) priority PAHs and of 29 PCBs (Table 2) was always below the limit of detection (LOD) of the method, namely 2 μg kg^−1^.

**Table 2:** List of polycyclic aromatic hydrocarbons (PAHs) and polychlorinated biphenyls (PCBs) investigated in the present study. PAH acronyms and PCB BZ congener numbers are reported in brackets.

*PAHs*			
Naphthalene	(NAP)	Benzo[a]anthracene	(B[a]A)
Acenaphthylene	(ACY)	Chrysene	(CHRY)
Acenaphthene	(ACE)	Benzo[b]fluoranthene	(B[b]F)
Fluorene	(FLU)	Benzo[k]fluoranthene	(B[k]F)
Phenanthrene	(PHEN)	Benzo[a]pyrene	(B[a]P)
Anthracene	(ANTH)	Benzo[g,h,i]perylene	(B[ghi]P)
Fluoranthene	(FLTH)	Indeno[1,2,3-c,d]pyrene	(IND)
Pyrene	(PYR)	Dibenz[a,h]anthracene	(D[ah]A)
*PCBs*			
2,4,4′-Trichlorobiphenyl	(28)	2,2′,3,4′,5,5′-Hexachlorobiphenyl	(146)
2,2′,5,5′-Tetrachlorobiphenyl	(52)	2,2′,3,4′,5′,6-Hexachlorobiphenyl	(149)
3,3′,4,4′-Tetrachlorobiphenyl	(77)	2,2′,3,5,5′,6-Hexachlorobiphenyl	(151)
3,4,4′,5-Tetrachlorobiphenyl	(81)	2,2′,4,4′,5,5′-Hexachlorobiphenyl	(153)
2,2′,3,5′,6-Pentachlorobiphenyl	(95)	2,3,3′,4,4′,5-Hexachlorobiphenyl	(156)
2,2′,4,4′,5-Pentachlorobiphenyl	(99)	2,3,3′,4,4′,5′-Hexachlorobiphenyl	(157)
2,2′,4,5,5′-Pentachlorobiphenyl	(101)	2,3′,4,4′,5,5′-Hexachlorobiphenyl	(167)
2,3,3′,4,4′-Pentachlorobiphenyl	(105)	3,3′,4,4′,5,5′-Hexachlorobiphenyl	(169)
2,3,3′,4′,6-Pentachlorobiphenyl	(110)	2,2′,3,3′,4,4′,5-Heptachlorobiphenyl	(170)
2,3,4,4′,5-Pentachlorobiphenyl	(114)	2,2′,3,3′,4,5′,6′-Heptachlorobiphenyl	(177)
2,3′,4,4′,5-Pentachlorobiphenyl	(118)	2,2′,3,4,4′,5,5′-Heptachlorobiphenyl	(180)
2,3′,4,4′,5′-Pentachlorobiphenyl	(123)	2,2′,3,4,4′,5′,6-Heptachlorobiphenyl	(183)
3,3′,4,4′,5-Pentachlorobiphenyl	(126)	2,2′,3,4′,5,5′,6-Heptachlorobiphenyl	(187)
2,2′,3,3′,4,4′-Hexachlorobiphenyl	(128)	2,3,3′,4,4′,5,5′-Heptachlorobiphenyl	(189)
2,2′,3,4,4′,5′-Hexachlorobiphenyl	(138)		

The concentration and occurrence of the heavy metals investigated was extremely variable; in particular, some of them (i.e., Be, CrVI, Sb, Sn, Tl and V) were always below the limit of quantification (LOQ), i.e., 10 μg kg^−1^, while others occurred mainly or exclusively in one species (Table 3). In detail, As, Co and Hg (7.24, 0.16 and 1.49 mg kg^−1^, respectively; average values from two samples) were found only in sperm whale faecal samples, while Pb (65 µg kg^−1^) only in faecal samples from fin whales. The average concentrations of Cd and Se in sperm whale faecal samples (0.45 and 10.6 mg kg^−1^, respectively) were one order of magnitude higher than in fin whale faecal samples, while the average concentration of Zn (97 mg kg^−1^) was 1.5–2 × higher (Table 3). On the opposite, fin whale faecal samples showed Cu and Ni average concentrations (61.3 and 1.14 mg kg^−1^, respectively) twofold and threefold compared to sperm whale faecal samples (Table 3).

**Table 3:** Average metal concentration (mg kg^−1^ dry matter) in faecal samples from fin and sperm whales. The concentration of some of these metals in seawater (μg L^−1^) and in *Meganyctiphanes norvegica* (mg kg^−1^ dry matter) have been reported only for comparison.

Sample	As	Be	Cd	Co	Cr_tot_	CrVI	Cu	Hg	Ni	Pb	Sb	Se	Sn	Tl	V	Zn
#1 (fin whale)	< LOQ	< LOQ	0.031	< LOQ	1.165	< LOQ	61.63	< LOQ	1.211	0.067	< LOQ	0.847	< LOQ	< LOQ	< LOQ	43.73
#2 (fin whale)	< LOQ	< LOQ	0.043	< LOQ	0.885	< LOQ	61.01	< LOQ	1.077	0.064	< LOQ	1.273	< LOQ	< LOQ	< LOQ	60.62
#3 (sperm whale)	7.139	< LOQ	0.413	0.169	0.915	< LOQ	34.93	1.42	0.352	< LOQ	< LOQ	11.01	< LOQ	< LOQ	< LOQ	95.71
#4 (sperm whale)	7.340	< LOQ	0.482	0.146	1.085	< LOQ	32.88	1.56	0.397	< LOQ	< LOQ	10.24	< LOQ	< LOQ	< LOQ	98.12
Seawater (μg L^−1^)^†^	3.7	0.0056	0.11	0.02	0.3	–	0.25	0.03	0.56	0.03	0.24	0.2	0.004	0.019	2.5	4.9
Seawater (μg L^−1^)^‡^	1.5	–	0.01	0.05	0.3	–	0.10	–	0.20	0.03	0.24	–	–	–	2.5	0.1
*M. norvegica* (mg kg^−1^)*	–	–	1.3	–	–	–	65.60	–	–	–	–	–	–	–	–	85.0
*M. norvegica* (mg kg^−1^)**	–	–	0.119	–	–	–	55.84	0.141	–	0.496	–	–	–	–	–	94.1

LOQ = limit of quantification (0.01 mg kg^−1^).

^†^Data from Bowen (1979).

^‡^Data from Chester (2000).

*Data from Fowler^52^.

**Data from Fossi et al.^53^, modified.

## Discussion

Fin and sperm whales residing or circulating in the Mediterranean Sea are exposed to biological and chemical hazard due to the increasing anthropogenic impact. In particular, most of the coastal areas bordering with the Sanctuary is heavily populated and full of commercial, touristic and military ports and industrial areas. As a consequence, a range of diverse human activities exerts several actual and potential threats to cetacean populations in the Sanctuary, including habitat degradation, urban, tourist, industrial, and agricultural development, intense maritime traffic, military exercises and oil and gas exploration, just to mention the most important ones.

This study provides background information on the occurrence and concentration of parasites and bacterial infections/communities as well a first investigation of heavy metals and organic pollutants in faecal samples from fin and sperm whale Mediterranean subpopulations within the Pelagos Sanctuary.

Here, a modified MINI-FLOTAC technique in combination with FILL-FLOTAC were used for parasitological detection of the cysts in the faecal samples of fin and sperm whales. Although this technique has never been used before for whale faecal samples, it has successfully been used in previous coprological surveys for the detection of gastrointestinal parasites in other marine animals as the loggerhead sea turtles (*Caretta caretta*)^23,24^. The MINI-FLOTAC can be considered as one of the most accurate methods for coprological diagnosis of endoparasite infections and cysts/eggs counting nowadays available in veterinary medicine^25^. It allowed an accurate and reliable detection of *Blastocystis* cysts in both fin and sperm faecal samples. Molecular analysis, sequencing and phylogenetic analysis confirmed the obtained results.

*Blastocystis* is a common intestinal protozoan parasite reported in several animals, e.g., humans, livestock, dogs, amphibians, reptiles, birds and even insects^26–28^. Although it possesses pathogenic potential, its virulence mechanisms in humans are still not well understood^29^. *Blastocystis* seems to be linked to Irritable Bowel Syndrome, i.e., a functional disorder mainly consisting in chronic or recurrent abdominal pain due to altered intestinal habits^30^. Studying the small subunit ribosomal RNA (*SSU-rDNA*) gene, several authors identified at least 22 different *Blastocystis* subtypes (ST) in a variety of animals, humans included, i.e., from ST1 to ST17, ST21, and ST23 to ST26 (Ref.^26^). To date, human *Blastocystis* isolates are classified into 10 ST (i.e., ST1-ST9 and ST12) that, with the only exception of ST9, have been identified also in other animals^31^. According to Parkar et al.^32^, *Blastocystis* has the potential to spread through human-to-human, animal-to-human, and human-to-animal contact.

Few similar parasitological investigations have been conducted in the past and are currently available in the literature. Hermosilla et al.^33^ detected three protozoan parasites (i.e., *Giardia* sp., *Balantidium* sp., *Entamoeba* sp.) and helminth parasites in individual faecal samples from wild fin (n. 10), sperm (n. 4), blue (*Balaenoptera musculus*; n. 2) and sei (*Balaenoptera borealis*; n. 1) Atlantic whale subpopulations from the Azores Islands, Portugal. Protozoan parasites (*Giardia* sp., *Balantidium* sp., *Cistoisospora*-like indet.) and helminth parasites were also found in individual faecal samples of wild sperm whales inhabiting Mediterranean Sea waters surrounding the Balearic Archipelago, Spain^34^. Out of these, three of herein detected parasites clearly bear anthropozoonotic potential, i.e., *Anisakis*, *Balantidium* and *Giardia*^34^.

In the present work, *Blastocystis* has been found in fin and sperm whale samples and, to the best of our knowledge, this is the first time that this protozoan genus is reported for any cetacean species. Therefore, this finding represents the first new host record for fin and sperm whales. *Blastocystis* ST3 was the only subtype found in fin and sperm whales. Molecular studies in human samples showed the occurrence of ST1–ST9, with ST3 as the most prevalent subtype^35,36^. Indeed, ST3 is the *Blastocystis* subtype with the highest prevalence in humans worldwide and probably represents the human species-specific ST (Ref.^37^). Consequently, animals harbouring ST3 may thus mirror environmental contamination by humans, confirming the zoonotic potential of animals for *Blastocystis* human infections. Unlike^33,34^, no eggs of helminths were found in our faecal samples.

Variations in parasites composition and prevalence might be related to several factors such as dietary differences, the parasite life cycle, the availability of hosts necessary to complete their life cycle, the interactions between parasite species, the host immune response, and the host population density^23^. Moreover, parasites can also spread in different way in animal populations in the wild, particularly when they act together with ecological, biological, and anthropogenic factors^38^.

The occurrence in whales of parasites with a zoonotic potential like *Giardia* or *Balantidium*, most probably due to coastal waters contaminated by sewage, agricultural and urban run-off, has been already reported elsewhere^39–43^. Furthermore, human excretions from increasing number of pleasure boats, fishing and whale watching boats could be an additional form of contamination. Finally, the intense maritime traffic in the Mediterranean Sea, the percentage of which is higher than in other oceans^44^, represents another source of contamination. In all cases, results highlight that human activities play an important role for the widespread of these pathogens.

No bacterial pathogen of human or terrestrial animal origin has been detected both by targeted PCR and by Illumina high throughput sequencing. This difference could be due to the lower survival rate of bacteria in the sea environment, compared to protozoan parasites^45^.

Previous works reported the occurrence of human pathogens in stranded common minke whale (*Balaenoptera acutorostrata*) from Philippines^46^ and killer whale respiratory microbiome in North Pacific^47^. Although the relatively low number of samples cannot exclude potential risk of transmission of human and zoonotic pathogenic bacteria to cetaceans in the surveyed area, our results suggest to focus on microbiological analyses to track potential internal waterborne pathogens to the ones able to form cysts (like parasites) or other forms of resistance (like spore-forming bacteria) that are more likely to survive for longer period in the seawater.

The dominance of Bacterioidetes and Firmicutes (common with other terrestrial mammals), the baleen-specific higher number of *Spirochaetes* and the lower of *Proteobacteria* characterized both species, as also reported elsewhere^21^. Moreover, differences of some taxa related with the diverse diet were confirmed: in the case of sperm whale, whose nutrition is based on cephalopods, a higher proportion of Synergistetes was observed in faecal samples, whereas faecal samples from fin whales had a higher level of *Spirochaetes* compared those from sperm whales. These findings are in agreement with Erwin et al.^13^. The Synergistetes phylum includes gram negative, anaerobic, rod-shaped bacteria, widely distributed in terrestrial and aquatic environments, including host-associated with mammals^48^. Within this phylum, *Synergistaceae* family and *Pyramidobacter* genus OTUs were particularly dominant among sperm whale microbiome (Fig. 4). However, no correlation with potential pathogenicity could be drawn from the presence of these specific OTUs, considering their ubiquity in oral and gut mucosa of marine and terrestrial animals, despite some of the genus belonging to *Synergistaceae* family (e.g. *Cloacibacillus* spp.) are considered opportunistic pathogens^49^. About potential health implication of Spirochaetes, similar conclusion than for Synergistetes could be drawn: *Treponema* sp., found as dominant genus in fin whales, were found in healthy baleen whales by Sanders et al.^21^ so as among more dynamical OTU in stranded right whales^50^ *Sphaerochaeta* spp. associated with healthy cetacean monitored oral cavity microbiome^14^. Moreover, fin whale faeces also showed a higher proportion of taxa that are also enriched in terrestrial herbivores, like Lentisphaere, Verrucomicrobia, Actinobacteria and Tenericutes, as also reported elsewhere^21^. Although differences in species sampled and habitats compared to previous studies, we found confirmation of both species and diet-influenced gut microbiota composition. Notably *Akkermansia* (one of the dominant Verrucomicrobia OTUs) and *Coriobacteriaceae* (dominant family among Actinobacteria phylum) includes typical holobiont of terrestrial and marine mammals, but also some pathobiont, so far confirmed only for humans^51^. Due to the wide distribution of some of Synergistetes and Spirochaetes phyla, it is not possible to establish if their presence could be ascribed exclusively to an anthropogenic impact; however, it is worth of interest that some of the genus found in both whale species and belonging to these phyla include opportunistic pathogens whose virulence for marine mammals still need to be confirmed. Interestingly, some archaeal sequences related to the Thermoplasmatales order were also found. This confirms what already reported by Sanders et al.^21^, i.e., that archaea belonging to this order may have a role as methane producer from methylated amines in baleen gut, differently from methanogenic archaea belonging to other orders that typically colonize the gut of terrestrial mammals, including humans.

Therefore, the two sampled species harboured typical gut microbiome belonging to fin whale and sperm whale groups. These data extend the spectrum of surveyed whales gut microbiome to previously unsampled species and confirms that NGS analyses could be a useful tool to retrieve information on the health status of wild whales.

While the concentration of 16 U.S. EPA priority PAHs and of 29 PCBs, being always < LOD (Table 2), did not provide useful information, the concentration and occurrence of some heavy metals was extremely useful to speculate about their background values as well as their potential as a proxy to distinguish between the two whale species. In fact, data reported in Table 3 clearly underline that As, Co and Hg were found only in sperm whale faecal samples, while Pb only in fin whale faeces.

Fowler^52^ demonstrated a high variability in the concentrations of many of these trace elements in several pelagic organisms from the open Mediterranean Sea. Monitoring the concentration of a suite of biomarkers, organochlorines, PAHs and heavy metals in *Meganyctiphanes norvegica*, the most abundant euphausiid in the western Mediterranean and the main constituent of Mediterranean krill, Fossi et al.^53^ hypothesized its potential utilization as a tool for the assessment of the health status in the Pelagos Sanctuary area in general, and of the *B. physalus* population (i.e., the main *M. norvegica* ‘predator’) in particular. In detail, the authors reported mean concentrations of 0.141, 0.119, 0.496 mg kg^−1^ for Hg, Cd and Pb, respectively (Table 3), carcinogenic PAHs ranging from 60.3 to 141.7 µg kg^−1^, and PCBs ranging from 84.6 to 210.2 µg kg^−1^. The highest values were generally detected in the station closest to the Ligurian coast.

On the opposite, cephalopods belonging to the *Histioteuthidae* family represent the main diet of sperm whales^54,55^ and to follow those belonging to the *Architeuthis* genus. Interestingly, Bustamante et al.^56^, have found high concentrations of Cd, Co, Cu and Se bioaccumulated in the digestive gland of *Architeuthis dux* from the Mediterranean and Atlantic Spanish waters, whereas high concentrations of As, Co, Hg, Ni, and Se were also found in branchial hearts.

Therefore, the occurrence of a metal exclusively in faeces from one whale species (i.e., As, Co and Hg detected only in sperm whales, and Pb, detected only in fin whale), and/or significant differences in the concentration of other metals (i.e., Cd, Cu, Ni, Se and Zn), may mirror their diverse diet (krill vs. cephalopods), as also suggested by elemental, coprological and microbiological analyses, and, in turn, the bioaccumulation potential of specific heavy metals through the different diet. The absence of PAHs and PCBs in faeces samples is probably due to their lipophilicity; as a consequence, ingestion of these organic pollutants by animals leads to bioaccumulation generally in the fatty tissues rather than their discharge throughout faeces. Considering the relatively low number of samples surveyed in the present work we cannot exclude that organic pollutants are present in the free living whales of the area; therefore the use of faecal samples as an indicator of PAHs and PCBs remains an open question needed to be further investigated.

## Conclusions

The present study confirms that fin and sperm whale Mediterranean subpopulations are exposed to anthropogenic pressure, emphasizing the relevance of constant surveillance of marine mammals to prevent pathogens transmission to humans and vice versa, and exposition to chemical pollutants. Among microbial and parasitological health risk, the latter seems to be more relevant in the investigated individuals, and different species may be exposed to specific chemical pollutants and opportunistic pathogens, according to their diet.

Considering the possibility of collecting in an easy way and without any disturb to the animals, the use of a faecal sample as a proxy of anthropogenic pressure has proven to be a valid indicator at least for pathogens and heavy metals.

New insights into these topics in whale populations and other marine animals in the wild will contribute to a better understanding of human-related impacts on marine ecosystem health and to the development of proper conservation tools.

In conclusion, this survey clearly provides baseline data on occurrence and background concentration of a new anthropozoonotic parasite, bacterial communities and heavy metals in free-ranging fin and sperm whales, and calls for more integrated research to perform regular monitoring programs supported by national and/or international authorities responsible for preservation of these still vulnerable and threatened whale species in the Mediterranean Sea.

## Material and methods

### Study area

The Pelagos Sanctuary is a marine protected area extending > 87.500 km^2^ in the Northern-Western Mediterranean Sea between the Italian peninsula, France and the Island of Sardinia, encompassing Corsica and the Tuscan Archipelago (Fig. 1)^57^. The Sanctuary waters include the Liguria Sea and parts of the Corsican and Tyrrhenian Seas, and contain the internal maritime (15%) and territorial waters (32%) of France, Monaco and Italy, as well as the adjacent high seas (53%). Within the Sanctuary area the continental shelf is wide only in correspondence of such limited coastal plains, whereas it is mostly narrow and disseminated with steep, deeply-cut submarine canyons elsewhere^57^.

High levels of primary production, with chlorophyll concentrations exceeding 10 g m^−3^ (Ref.^58,59^), support a conspicuous biomass of highly diversified zooplankton fauna, including gelatinous macro zooplankton and swarming euphausiid crustaceans *M. norvegica* (krill)^57^ and cephalopods belonging mainly to Histioteuthidae family^54,55^ and to *Architeuthis* genus. Zooplankton, in turn, attracts to the area a various level of predators, cetaceans included.

### Sampling

In the framework of a research project on the ecology of whales, faecal samples were collected into the Pelagos Sanctuary (Fig. 1) from sperm and fin whales (Table 1).

During the summer boat survey, photo identification and floating faeces were collected from individual whales using a fine nylon mesh net, avoiding direct contacts with animals. Faecal samples were immediately placed in sterile falcon, labelled for whale identification and stored for further parasitological, bacteriological and chemical analyses.

### Coprological analyses

Each faecal sample was subjected to microscopic investigation by using a flotation solution of ZnSO_4_ (specific gravity 1360) and a MINI-FLOTAC technique in combination with FILL-FLOTAC slightly modified as reported elsewhere^24^.

### Molecular analyses

#### DNA extraction, PCR amplification and sequencing

Genomic DNA was extracted from each faecal sample of approximately 200 mg by using the QiaAMP DNA Stool Mini Kit (Qiagen, Hilden, Germany) according to the manufacturer instructions and then eluted in 200 μL TE buffer.

Primers RD5 (5′-ATCTGGTTGATCCTGCCAGT-3′) and BhRDr (5′-GAGCTTTTTAACTGCAACAACG-3′) were used to amplify approximately a fragment of 600 base pair within the 1800 bp *SSU-rDNA* of *Blastocystis*^60^. The PCR reaction mix contained 10 μL of Phire Reaction Buffer 5 × (Thermo Scientific, USA), 0.4 μL dNTPs (200 μM) (Qiagen, USA), 1 μL primer pairs (10 μM), 0.4 μL of Phire Hot Start II DNA Polymerase 1U (Thermo Scientific, USA) and 5 μL (approximately 100 ng) genomic DNA per reaction according the manufacturer’s protocol. The PCR protocol was as follows: denaturation at 98 °C for 30 s, followed by 98° C for 5 s, 59 °C for 30 s and 72 °C for 1 min for 35 cycles, and finally 72 °C for 2 min. Negative controls (PCR quality water) were included in each PCR run.

The PCR fragments were run on 1.2% agarose gel and positive samples were purified with Exonuclease I (EXO I) and Thermosensitive Alkaline Phosphatase (FAST AP) (Fermentas) enzymes according the manufacturer instructions^61^.

Purified amplicons were directly sequenced in both directions using the ABI PRIMS Big Dye Terminator v. 3.1 Cycle Sequencing Kit (Applied Biosystems, Foster City, California, USA) with the same primers as the respective PCR reaction, according to the manufacturer instructions. Sequences obtained were determined on an ABI PRISM 3130 Genetic Analyzer (Applied Biosystems, USA) and the chromatograms were inspected by eye using the Finch TV software. Primer regions plus bad-quality regions were removed^61^.

Once the sequences had been cleaned up, each sequence was compared with the *Blastocystis* homologous nucleotide sequences available in GenBank databases using the Blastn program (https://blast.ncbi.nlm.nih.gov). Then, the obtained sequences corresponding to *Blastocystis* *SSU-rDNA* gene portion were gathered in a fasta file and aligned each other using the ClustalW implementation of the BioEdit software v7.0.5, and the alignment was adjusted manually, when necessary^61^. To attribute the subtypes, a phylogenetic analysis of the obtained sequences and the homologous sequences from GenBank representing the *SSU-rDNA* *Blastocystis* subtypes ST1–17 were performed using the maximum likelihood method in Mega v7.0.9. The tree was rooted using a *Blastocystis lapemi* sequence as the outgroup (accession number: AY590115). Bootstrap confidence values for the branching reliability were calculated with 10,000 replicates.

### Microbiological analyses

The detection of pathogenic bacteria *Salmonella* spp. and *E. coli* O157:H7 were performed by duplex PCR analyses targeting respectively *invA* and *OriC* genes for *Salmonella*^62,63^ and *Rfb* and *fliC* genes for *E. coli* O157:H7 (Ref.^64^). PCR protocols used have already been described in detail in a previous work^65^.

DNA samples were sent to Stab Vida Lda. (Caparica – Portugal) for amplification, library construction and multiplexed sequencing of partial (V3-V4) 16S rRNA gene sequences on an Illumina MiSeq platform. Specifically, the library construction was performed using the Illumina 16S Metagenomic Sequencing Library preparation protocol. The generated DNA fragments were sequenced with MiSeq Reagent Kit v3, using 300 bp paired-end sequencing reads. The generated raw sequence data were analysed by using QIIME2 v2018.6.0 (Ref.^66^). The reads were denoised using the DADA2 plugin^67^. After denoising a total of 1142 unique features (OTUs) were identified. The scikit-learn classifier was used^68^ to train the classifier using the SILVA (release 132 QIIME) database, with a clustering threshold of 97% similarity. For classification purposes, only OTUs containing at least 10 sequence reads were considered as significant.

### Chemical analyses

Total carbon, nitrogen and sulphur concentrations were determined by flash combustion using an Elemental Analyser (CHNS vario Macro Cube, Elementar, Germany). Sulfanilic acid was used as a standard. Samples were analysed in duplicate and the coefficient of variation for all elements was always < 2%.

The concentration of 15 heavy metals (i.e., As, Be, Cd, Co, Cr, Cu, Hg, Ni, Pb, Sb, Se, Sn, Tl, V, Zn) was determined by Inductively Coupled Plasma–Mass Spectrometry (ICP-MS; Agilent 7800) after digestion with nitric and hydrochloric acid. The limit of quantification (LOQ) was 10 μg kg^−1^. The concentration of CrVI was determined through the alkaline digestion of samples (U.S. EPA Method 3060A)^69^ followed by the colorimetric assay with diphenylcarbazide (U.S. EPA Method 7196A)^70^.

The concentration of 16 U.S. EPA priority PAHs and 29 PCBs was determined following EPA protocols (U.S. EPA Method 3550C and 8270D, respectively)^71,72^ and analysed by gas chromatography equipped with a mass spectrometry detector (GC–MS). GC–MS analysis was performed with an Agilent Technologies gas chromatograph 7820A series equipped with a mass detector MSD Agilent Technologies 5977 series and the data analysis station ChemStation. A HP5MS UI column (30 m × 0.25 mm I.D. × 0.25 µm film thickness, Agilent Technologies) was used for the analysis. High-grade helium, at a constant flow rate of 1.0 mL min^−1^, was used as carrier gas. The GC–MS oven temperature program started at 50 °C and then increased to 280 °C at a rate of 20 °C min^−1^; this temperature was maintained for 5 min, increased at a rate of 5 °C min^−1^ until 340 °C, and held for 15 min. All analyses were carried out in selected ion monitoring (SIM) mode. The limit of detection (LOD) for each PAHs and PCBs was 2 μg kg^−1^.

### Ethics statement

All experiments were performed in accordance with relevant guidelines and regulations, and all protocols were approved by the Ethical Committee of the Department of the Sciences of Agriculture, Food, Natural Resources and Engineering, University of Foggia.

## Data Availability

Data related to this study are available from the corresponding author upon request.
